# Amplitude-modulated electromagnetic fields for the treatment of cancer: Discovery of tumor-specific frequencies and assessment of a novel therapeutic approach

**DOI:** 10.1186/1756-9966-28-51

**Published:** 2009-04-14

**Authors:** Alexandre Barbault, Frederico P Costa, Brad Bottger, Reginald F Munden, Fin Bomholt, Niels Kuster, Boris Pasche

**Affiliations:** 1 Cabinet Médical, Avenue de la Gare 6, Lausanne, Switzerland; 2 Rue de Verdun 20, Colmar, France; 3 Sirio-Libanes Hospital, Oncology Center, São Paulo, Brazil; 4 Radiology Associates, Danbury Hospital, Danbury, CT, USA; 5 Department of Radiology, The University of Alabama at Birmingham and UAB Comprehensive Cancer Center, Birmingham, AL, USA; 6 SPEAG AG, Zurich, Switzerland; 7 IT'IS, Swiss Federal Institute of Technology, Zurich, Switzerland; 8 Division of Hematology/Oncology, Department of Medicine, The University of Alabama at Birmingham and UAB Comprehensive Cancer Center, Birmingham, AL, USA

## Abstract

**Purpose:**

Because *in vitro *studies suggest that low levels of electromagnetic fields may modify cancer cell growth, we hypothesized that systemic delivery of a combination of tumor-specific frequencies may have a therapeutic effect. We undertook this study to identify tumor-specific frequencies and test the feasibility of administering such frequencies to patients with advanced cancer.

**Patients and methods:**

We examined patients with various types of cancer using a noninvasive biofeedback method to identify tumor-specific frequencies. We offered compassionate treatment to some patients with advanced cancer and limited therapeutic options.

**Results:**

We examined a total of 163 patients with a diagnosis of cancer and identified a total of 1524 frequencies ranging from 0.1 Hz to 114 kHz. Most frequencies (57–92%) were specific for a single tumor type. Compassionate treatment with tumor-specific frequencies was offered to 28 patients. Three patients experienced grade 1 fatigue during or immediately after treatment. There were no NCI grade 2, 3 or 4 toxicities. Thirteen patients were evaluable for response. One patient with hormone-refractory breast cancer metastatic to the adrenal gland and bones had a complete response lasting 11 months. One patient with hormone-refractory breast cancer metastatic to liver and bones had a partial response lasting 13.5 months. Four patients had stable disease lasting for +34.1 months (thyroid cancer metastatic to lung), 5.1 months (non-small cell lung cancer), 4.1 months (pancreatic cancer metastatic to liver) and 4.0 months (leiomyosarcoma metastatic to liver).

**Conclusion:**

Cancer-related frequencies appear to be tumor-specific and treatment with tumor-specific frequencies is feasible, well tolerated and may have biological efficacy in patients with advanced cancer.

**Trial registration:**

clinicaltrials.gov identifier NCT00805337

## Background

We have previously shown that the intrabuccal administration of low and safe levels of electromagnetic fields, amplitude-modulated at a frequency of 42.7 Hz by means of a battery-powered portable device modifies the electroencephalographic activity of healthy subjects [1,2], and is associated with subjective and objective relaxation effects [3]. We have also shown that sequential administration of four insomnia-specific frequencies, including 42.7 Hz, results in a significant decrease in sleep latency and a significant increase in total sleep time in patients suffering from chronic insomnia [4,5]. This approach has been termed Low Energy Emission Therapy (LEET)[4]. Dosimetric studies have shown that the amount of electromagnetic fields delivered to the brain with this approach is 100 to 1000 times lower than the amount of electromagnetic fields delivered by handheld cellular phones and does not result in any heating effect within the brain [6]. The U.S. FDA has determined that such a device is not a significant risk device. A long-term follow-up survey of 807 patients who have received this therapy in the U.S., Europe and Asia revealed that the rate of adverse reactions were low and were not associated with increases in the incidence of malignancy or coronary heart disease [7].

While many discoveries in medicine have evolved from a scientific rationale based on *in vitro *and *in vivo *findings, several seminal discoveries are the results of biological effects first observed in humans. For example, the development of modern cancer chemotherapy can be traced directly to the clinical observation that individuals exposed to mustard gas, a chemical warfare agent, had profound lymphoid and myeloid suppression. These observations led Goodman and Gilman to use this agent to treat cancer[8]. Given the advantageous safety profile of athermal, non-ionizing radiofrequency electromagnetic fields[7] and the emerging evidence that low levels of electromagnetic or electric fields may modify the growth of tumor cells [9-11], we hypothesized that the growth of human tumors might be sensitive to different but specific modulation frequencies. We tested this hypothesis through examination of a large number of patients with biopsy-proven cancer. Using a patient-based biofeedback approach we identified strikingly similar frequencies among patients with the same type of cancer and observed that patients with a different type of cancer had biofeedback responses to different frequencies. These findings provided strong support for our initial hypothesis. Following identification of tumor-specific frequencies in 163 patients with a diagnosis of cancer, we offered compassionate treatment to 28 patients with advanced cancer and limited palliative therapeutic options. We are reporting the results of our frequency discovery studies as well as the results of a feasibility study making use of Low Energy Emission Therapy in the treatment of cancer.

## Methods

Frequency discovery consists in the measurement of variations in skin electrical resistance, pulse amplitude and blood pressure. These measurements are conducted while individuals are exposed to low and safe levels of amplitude-modulated frequencies emitted by handheld devices. Exposure to these frequencies results in minimal absorption by the human body, which is well below international electromagnetic safety limits [12,13]. Patients are lying on their back and are exposed to modulation frequencies generated by a frequency synthesizer as described below. Variations in the amplitude of the radial pulse were used as the primary method for frequency detection. They were defined as an increase in the amplitude of the pulse for one or more beats during scanning of frequencies from 0.1 to 114,000 Hz using increments of 100 Hz. Whenever a change in the amplitude of the pulse is observed, scanning is repeated using increasingly smaller steps, down to 10^-3 ^Hz. Frequencies eliciting the best biofeedback responses, defined by the magnitude of increased amplitude and/or the number of beats with increased amplitude, were selected as tumor-specific frequencies.

**Figure 1:**
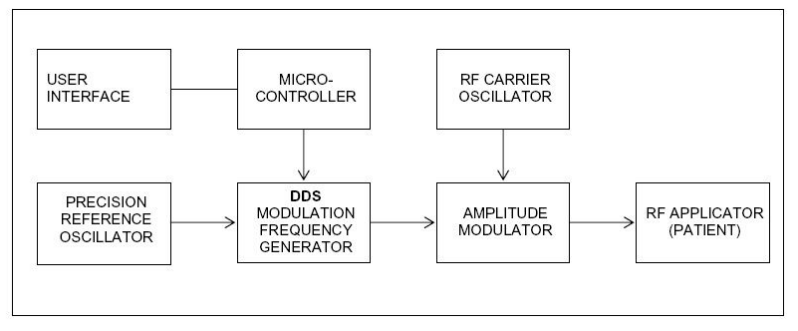
**Block diagram of the novel emitting device making use of the Direct Digital Synthesis (DDS) technology **. This applicator was used for both the detection and administration of amplitude-modulated electromagnetic frequencies. RF: radiofrequency.

During our initial search for frequencies in patients with a diagnosis of cancer, we identified frequencies in the 1,000 to 15,000 Hz range. The range of these frequencies was higher than the frequencies previously identified in patients with insomnia (< 300 Hz). To enable the administration of well defined signals at these higher frequencies, the signal synthesizer used in the insomnia studies was redesigned and its accuracy verified at the laboratories of the Foundation for Research on Information Technology in Society (IT'IS, Zurich, Switzerland). The Direct Digital Synthesis (DDS) based synthesizer AD9835 (Analog Devices, Norwood, MA) with a frequency precision of 10^-7 ^was used for frequency detection in patients with a diagnosis of cancer. Subsequently, the same frequency synthesizer was used for treatment administration. The concept of this novel device is depicted in Figure 1.

Generation of amplitude-modulated electromagnetic fields: the device consists of a battery-driven radiofrequency (RF) electromagnetic field generator connected to a 1.5 meter long 50 Ohm coaxial cable, to the other end of which a spoon-shaped mouthpiece made of steel is connected with the inner conductor. The RF source of the device corresponds to a high-level amplitude-modulated class C amplifier operating at 27.12 MHz. The modulation frequency can be varied between 0.01 Hz and 150 kHz with a modulation depth of 85 ± 5%. The output signal is controlled by a microcontroller AT89S8252 (Atmel, Fribourg, Switzerland), i.e. duration of a session, sequence of modulation frequencies, and duration of each sequence are programmed prior to the treatment with a PC connected to the panel of the device. The RF output is adjusted to 100 mW into a 50 Ohm load using a sinusoidal modulated test signal, which results in an emitting power identical to that of the device used in the treatment of insomnia [4,5].

### Compassionate treatment

Following a period of search and discovery of novel tumor-specific frequencies, outpatient treatment of patients with advanced cancer was initiated in Switzerland and Brazil on a compassionate basis, free of charge. Patients self-administered treatment for 60 min, three times a day. Oral informed consent was provided by seven patients. All other patients signed a written informed consent approved by a local human subject committee in compliance with the Helsinki declaration and the protocol was registered, clinicaltrials.gov identifier # NCT00805337. All patients had histologically confirmed diagnosis of cancer. Except for patients with a diagnosis of ovarian cancer, measurable disease was required. For patients with ovarian cancer, CA 125 level was used as a surrogate marker of measurable disease and a 50% increase in baseline level considered evidence of disease progression. All anticancer therapies had to be discontinued for at least one month prior to treatment initiation. Other eligibility criteria included an Eastern Cooperative Group performance status (PS) of 0 to 2 and an estimated life expectancy of at least 3 months.

### Disease assessment

Objective response in patients with measurable disease was assessed using the Response Evaluation Criteria in Solid Tumors group classification [14]. Two of us (B.B. and R.F.M.) independently reviewed all imaging studies.

### Toxicity assessment

Patients were evaluated for treatment-related toxicity at a minimum every two months as per the National Cancer Institute Common Toxicity Criteria version 2.0. The worst grade of toxicity per patient was recorded.

## Results

### Patients characteristics

A total of 115 patients were examined in Switzerland, 48 in Brazil (Table 1). There were 76 females and 87 males. The median age was 59 years (range 19 – 84). The most common tumor types were hepatocellular carcinoma (46), breast cancer (32), colorectal cancer (19), and prostate cancer (17).

**Table 1:** Frequency discovery in 163 patients with a diagnosis of cancer

**Tumor type**	**Number of patients**	**Number of frequency detection sessions**	**Number of frequencies**	**Tumor-specific frequencies** **Nb and (%)**	**Frequencies common to two or more tumor types**
**Brain tumors**	8	22	57	41 (71.9)	16
**Hematologic malignancies**	7	13	56	44 (78.6)	12
**Colorectal cancer**	19	40	99	67 (67.7)	32
**Hepatocellular carcinoma**	46	63	170	144 (84.7)	26
**Pancreatic cancer**	6	44	162	125 (77.2)	37
**Ovarian cancer**	10	66	278	219 (78.8)	59
**Breast cancer**	32	93	188	141 (75.0)	47
**Prostate cancer**	17	80	187	150 (80.2)	37
**Lung cancer**	6	17	80	57 (71.3)	23
**Renal cell cancer**	2	3	36	33 (91.7)	3
**Thyroid cancer**	1	14	112	89 (79.5)	23
**Neuroendocrine tumor**	5	5	30	17 (56.7)	13
**Bladder cancer**	2	4	31	25 (80.6)	6
**Leiomyosarcoma**	1	2	36	31 (86.1)	5
**Thymoma**	1	1	2	0 N/A	2
					
**Total**	163	467	1524	1183 (77.6)	341

The following frequencies were common to most patients with a diagnosis of breast cancer, hepatocellular carcinoma, prostate cancer and pancreatic cancer: 1873.477 Hz, 2221.323 Hz, 6350.333 Hz and 10456.383 Hz

Compassionate treatment with tumor-specific frequencies was offered to 28 patients (Table 2). Twenty six patients were treated in Switzerland and two patients were treated in Brazil. All patients were white, and 63% (n = 17) were female. Patients ranged in age from 30 to 82 years (median, 61 years) and 75% (n = 21) had PS of 1 (*vs *0 or 2). Seventy-nine percent (n = 22) of patients had received at least one prior systemic therapy, 57% (n = 17) had received at least two prior systemic therapies (Table 2).

**Table 2:** Characteristics of patients treated with amplitude-modulated electromagnetic fields

**Characteristic**	**No**	**%**
Age, years		
Median	61.0	
Range	30–82	
		
Sex		
Male	11	39.3
Female	17	60.7
		
Performance status, ECOG		
0	1	3.6
1	21	75.0
2	6	21.4
		
Sites of primary disease		
Breast	7	25.0
Ovary	5	17.9
Pancreas	3	10.7
Colon	2	7.1
Prostate	2	7.1
Glioblastoma multiforme	1	3.6
Hepatocellular carcinoma	1	3.6
Mesothelioma	1	3.6
Neuroendocrine	1	3.6
NSCLC	1	3.6
Oligodendroglioma	1	3.6
SCLC	1	3.6
Sarcoma	1	3.6
Thyroid	1	3.6
		
Prior systemic therapy		
Yes	22	78.6
No	6	21.4

Once disease progression was observed, most patients elected to resume or initiate chemotherapy and/or targeted therapy. Seven (25%) patients requested to continue experimental treatment in combination with chemotherapy. Continuation of experimental treatment was allowed if desired by the patient and approved by the patient's oncologist.

### Discovery of tumor-specific frequencies

The exact duration of each examination was not recorded but lasted on average three hours. Each patient was examined an average of 3.3 ± 3.4 times (range 1 – 26). Frequency discovery was performed in patients with disease progression, stable disease or partial response. In general, we found more frequencies in patients with evidence of disease progression and large tumor bulk than in patients with stable disease, small tumor bulk or evidence of response. When we restrict the analysis of patients examined in 2006 and 2007, i.e. at a time when we had gathered more than 80% of the common tumor frequencies, we found that patients with evidence of disease progression had positive biofeedback responses to 70% or more of the frequencies previously discovered in patients with the same disease. Conversely, patients with evidence of response to their current therapy had biofeedback responses to 20% or less of the frequencies previously discovered in patients with the same disease. We also observed that patients examined on repeated occasions developed biofeedback responses to an increasing number of tumor-specific frequencies over time whenever there was evidence of disease progression. Whenever feasible, all frequencies were individually retested at each frequency detection session. These findings suggest that a larger number of frequencies are identified at the time of disease progression.

A total of 1524 frequencies ranging from 0.1 to 114 kHz were identified during a total of 467 frequency detection sessions (Table 1). The number of frequencies identified in each tumor type ranges from two for thymoma to 278 for ovarian cancer. Overall, 1183 (77.6%) of these frequencies were tumor-specific, i.e. they were only identified in patients with the same tumor type. The proportion of tumor-specific frequencies ranged from 56.7% for neuroendocrine tumors to 91.7% for renal cell cancer. A total of 341 (22.4%) frequencies were common to at least two different tumor types. The number of frequencies identified was not proportional to either the total number of patients studied or the number of frequency detection sessions (Table 1).

### Treatment with tumor-specific amplitude-modulated electromagnetic fields

**Figure 2:**
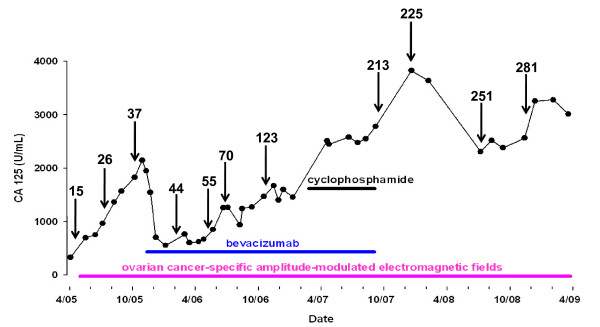
**Compassionate treatment of a 51 year old patient with ovarian cancer FIGO IIIC with extensive peritoneal carcinomatosis since October 1997**. The patient received paclitaxel and cisplatin from March 97, then docetaxel and carboplatin, doxorubicin, and gemcitabine. Because of progression of disease the patient was offered compassionate treatment with amplitude-modulated electromagnetic fields as of May 05. As seen below, the initial treatment consisting of 15 frequencies (May 05) did not yield any response. Upon re examination, 11 additional frequencies (26) were added to the treatment program in August 05. Because of disease progression, treatment with single agent bevacizumab was initiated in November 05. Interestingly, the CA 125 level had decreased by 200 units prior to the initiation of bevacizumab. Combined treatment with amplitude-modulated electromagnetic fields and bevacizumab resulted in a decrease in CA 125 level from 2140 to 540 in May 06. Treatment was supplemented with cyclophosphamide from March to September 07. The patient was hospitalized with pneumonia and elected to only receive amplitude-modulated electromagnetic fields since September 07. As of April 09, i.e. 50.5 months after treatment initiation the patient has stable disease and is asymptomatic. The numbers above the arrows represent the total number of cancer-specific frequencies included in the treatment program.

Twenty eight patients received a total of 278.4 months of experimental treatment. Median treatment duration was 4.1 months per patient; range 1 to +50.5. Patients treated in Switzerland were re-examined on average every other month for frequency detection; patients treated in Brazil were only examined once. Novel frequencies discovered upon re-examination were added to the treatment program of patients receiving experimental treatment. The first treatment programs consisted of combinations of less than ten frequencies while the most recent treatment programs exceed 280 frequencies (Figure 2).

The evolution of treatment programs through incremental addition of tumor-specific frequencies is illustrated by the case of a 51 year old woman with ovarian cancer. This patient was diagnosed with FIGO stage III (G2–G3) ovarian cancer in October 1997 and had received multiple courses of palliative chemotherapy until 2005. As seen on Figure 2, the initial treatment consisting of 15 frequencies did not yield any response. Upon re-examination, 11 additional frequencies (total of 26) were added to the treatment program in August 05. Because of disease progression, treatment with single agent bevacizumab was initiated in November 05. Interestingly, the CA 125 level had decreased by 200 units between October and November 2005, prior to the initiation of bevacizumab. Combined treatment with amplitude-modulated electromagnetic fields and bevacizumab resulted in a decrease in CA 125 level from 2140 to 540 in May 06. Treatment was supplemented with cyclophosphamide from March to September 07. The patient was hospitalized with pneumonia and elected to only receive amplitude-modulated electromagnetic fields since September 07. As of April 1, 2009 the patient has stable disease and is asymptomatic. She has been receiving experimental treatment without interruption for a total of +50.5 months.

**Figure 3:**
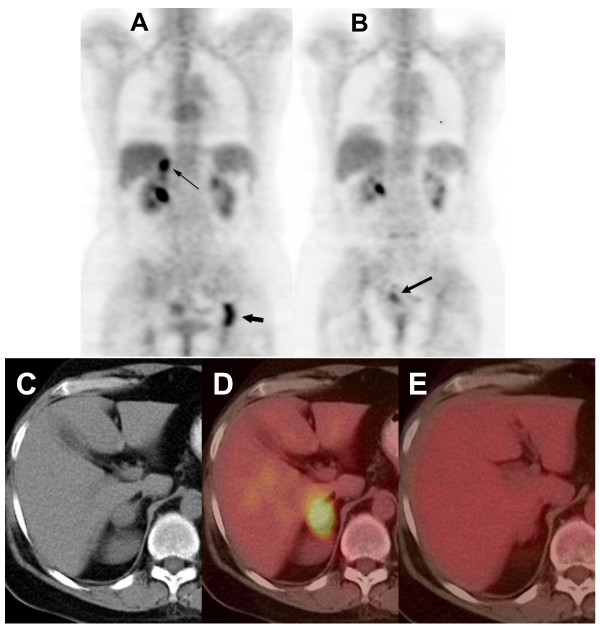
**59 yo postmenopausal female with ER/PR positive, ERBB2 negative breast cancer with biopsy confirmed metastasis to the left ischium and right adrenal gland**. A) Baseline PET MIP image demonstrates metastatic disease of the right adrenal gland (small arrow) and the left ischium (large arrow). B) PET MIP image four months after baseline shows the FDG activity in the right adrenal and left ischium has resolved indicating response to therapy. However, a primary uterine tumor, which was barely detectable in the baseline study, grew during the same time frame (arrow). C) Baseline PET/CT (left panel): The non-contrast CT shows an enlarged right adrenal gland. D) Baseline PET/CT (right panel): The fused PET/CT demonstrates increased FDG activity in the enlarged right adrenal gland. E) Follow-up PET/CT: The fused PET/CT four months after baseline shows a decrease in FDG activity of the right adrenal gland. Note the corresponding decrease in size also.

This case provides empirical evidence that adding tumor-specific frequencies may yield disease stabilization in patients with evidence of disease progression. However, addition of frequencies over time does not appear to be a requirement for therapeutic efficacy. This is illustrated by the case of a 59 yo postmenopausal female with ER/PR positive, ERBB2 negative breast cancer with biopsy confirmed metastasis to the left ischium and right adrenal gland (Figure 3A, Figure 3C, Figure 3D). She had been previously treated with radiation therapy to the left ischium, had received five different hormonal manipulations (tamoxifen, anastrozole, exemestane, fulvestran and megestrol). She had also received capecitabine, which had been discontinued because of gastrointestinal side effects. The patient was examined only once. In June 2006, at the time of treatment initiation, the patient complained of severe left hip pain, which was limiting her mobility despite the intake of opioids. Within two weeks of experimental treatment initiation with breast cancer-specific frequencies, the patient reported complete disappearance of her pain and discontinued the use of pain medications. She also reported a significant improvement in her overall condition. As seen on Figure 3B and 3E, PET-CT obtained three months after treatment initiation showed complete disappearance of the right adrenal and left ischium lesions. The complete response lasted 11 months. Intriguingly, the patient had developed intermittent vaginal spotting in the months preceding experimental treatment initiation. A minimally enhancing uterine lesion was observed on PET-CT prior to treatment initiation. Upon follow-up, FDG uptake increased significantly (Figure 3B) and the patient was diagnosed with uterine cancer by hysteroscopy. The patient underwent hysterectomy, which revealed endometrial adenocarcinoma. Hence, while treatment with breast cancer specific frequencies resulted in a complete response, it did not affect the growth of endometrial adenocarcinoma. This observation suggests that breast cancer frequencies are tumor-specific as a response of the metastatic breast cancer was observed while a uterine tumor progressed.

As seen in Table 3, sixteen patients were evaluable for response by RECIST criteria. A complete response was observed in a patient with hormone-refractory breast cancer metastatic to the adrenal gland and bone (Figure 3), which lasted 11 months. A partial response was observed in a patient with hormone-refractory breast cancer metastatic to bone and liver, which lasted 13.5 months. Five patients had stable disease for +34.1 months (thyroid cancer with biopsy-proven lung metastases), 6.0 months (mesothelioma metastatic to the abdomen), 5.1 months (non-small cell lung cancer), and 4.1 months (pancreatic cancer with biopsy-proven liver metastases). As of April 1, 2009 two patients are still receiving experimental treatment and four patients are alive.

**Table 3:** Independent review of best response (N = 16) according to RECIST criteria

**Best response**	**No**	**%**
Complete response*	1	3.6
Partial response**	1	3.6
Stable disease***	5	28.6
Progressive disease****	9	21.4
		
Not available for response assessment	12	60.7

* Duration of the complete response was 11 months (breast cancer metastatic to bone and adrenal gland)

** Duration of the partial response was 13.5 months (breast cancer metastatic to bone and liver)

*** Duration of stable disease was +34.1 months (thyroid cancer metastatic to lung), 6.0 months (mesothelioma metastatic to abdomen), 5.1 months (Non-small cell lung cancer), 4.1 months (pancreatic cancer metastatic to liver), and 4.0 months (leiomyosarcoma metastatic to liver).

**** One patient with ovarian cancer had progressive disease while receiving 26 frequencies. She has now stable disease and has been receiving amplitude-modulated electromagnetic fields for +50.5 months (Figure 2).

Not included is a patient with breast cancer metastatic to bone and liver with a near complete response who started systemic chemotherapy with docetaxel and bevacizumab within 4 weeks of experimental treatment initiation.

### Adverse and beneficial reactions

No patients receiving experimental therapy reported any side effect of significance and no patient discontinued treatment because of adverse effects. Three patients (10.7%) reported grade I fatigue after receiving treatment. One patient (3.6%) reported grade I mucositis after long-term use (26 months) of the experimental device and concomitant chemotherapy. Two patients with severe bony pain prior to initiation of experimental treatment reported significant symptomatic improvement. Both patients had breast cancer metastatic to the skeleton.

## Discussion

In this report we describe the discovery of tumor-specific amplitude modulation frequencies in patients with a diagnosis of cancer using noninvasive biodfeedback methods. Our approach represents a significant departure from the development of novel forms of chemotherapy and targeted therapy, which commonly rely on *in vitro *and animal experiments, followed by phase I studies to assess tolerability. Given the absence of theoretical health risks related to the administration of very low level of electromagnetic fields and the excellent safety profile observed in patients suffering from insomnia treated for up to several years [7], our approach was entirely patient-based. This allowed us to examine a large number of patients with tumor types commonly encountered in Switzerland and Brazil. It also allowed us to examine the same patients on multiple occasions, which decreased the variability inherent to a single frequency detection session.

Examination of patients with cancer led to the identification of frequencies that were either specific for a given tumor type or common to two or more tumor types. We observed that most frequencies were tumor-specific. Indeed, when the analysis of frequencies is restricted to tumor types analyzed following a minimum of 60 frequency detection sessions (breast cancer, hepatocellular carcinoma, ovarian cancer and prostate cancer), at least 75% of frequencies appear to be tumor-specific. Some frequencies such as 1873.477 Hz, 2221.323 Hz, 6350.333 Hz and 10456.383 Hz are common to the majority of patients with a diagnosis of breast cancer, hepatocellular carcinoma, prostate cancer and pancreatic cancer. The small number of frequency detection sessions conducted in patients with thymoma, leiomyosarcoma, and bladder cancer constitutes a limitation of our study and an accurate estimate of tumor-specific versus nonspecific frequencies cannot yet be provided for these tumor types. Only one patient with thyroid cancer metastatic to the lung was examined 14 times over the course of the past three years and this led to the discovery of 112 frequencies, 79.5% of which were thyroid cancer-specific. These combined findings strongly suggest that many tumor types have a proportion of tumor-specific frequencies of more than 55%. The high number of frequencies observed in patients with ovarian cancer may be due to the various histologies associated with this tumor type.

We observed excellent compliance with this novel treatment as patients were willing to self-administer experimental treatment several times a day. The only observed adverse effects in patients treated with tumor-specific frequencies were grade I fatigue after treatment (10.6%) and grade I mucositis (3.6%). Fatigue was short-lived and no patient reported persistent somnolence. Of note, mucositis only occurred concomitantly with the administration of chemotherapy. The frequency and severity of adverse effects is analogous to what was observed in patients treated with insomnia-specific frequencies [5] and confirm the feasibility of this therapeutic approach and its excellent tolerability.

We did not observe any untoward reaction in patient receiving either chemotherapy or targeted therapy in combination with amplitude-modulated electromagnetic fields. While these latter findings are limited to 7 patients, they are consistent with the lack of theoretical interaction between very low level of electromagnetic fields and anticancer therapy. Furthermore, one patient received palliative radiation therapy concomitantly with experimental therapy without any adverse effects. These findings provide preliminary data suggesting that amplitude-modulated electromagnetic fields may be added to existing anticancer therapeutic regimens.

The objective responses observed suggest that electromagnetic fields amplitude-modulated at tumor-specific frequencies may have a therapeutic effect. Of the seven patients with metastatic breast cancer, one had a complete response lasting 11 months, another one a partial response lasting 13.5 months. These data provide a strong rationale to further study this novel therapy in breast cancer. The increased knowledge of tumor-specific frequencies and the preliminary evidence that additional tumor-specific frequencies may yield a therapeutic benefit (Figure 2) provides a strong rationale for the novel concept that administration of a large number of tumor-specific frequencies obtained through the follow-up of numerous patients may result in long-term disease control. This hypothesis is partially supported by two long-term survivors reported in this study, a patient with thyroid cancer metastatic to the lung with stable disease for +34.1 months and a heavily pretreated patient with ovarian carcinoma and peritoneal carcinomatosis with stable disease for +50.5 months. Additional support for this hypothesis stems from the observation that four patients with advanced hepatocellular carcinoma in a follow-up phase II study by Costa et al had a partial response, two of them lasting more than 35 months[15]. These exciting results provide hope that this novel therapeutic approach may yield long-term disease control of advanced cancer.

Kirson et al have recently reported the use of continuous wave (CW) electric fields between 100 KHz to 1 MHz [10,11]. These fields were CW, applied at relative high field strengths but lower frequencies than the fields used in our study. These frequencies were found to be effective when applied by insulating external electrodes to animal cancer models and patients with recurrent glioblastoma. In contrast to our approach, the electric fields applied to cancer cells and patients did not include any amplitude modulation. Hence, it is likely that these two different therapeutic modalities have different mechanisms of action.

Computer simulation studies have shown that the specific absorption rate (SAR) in the head resulting from the use of intrabuccally-administered amplitude-modulated electromagnetic fields is in the range of 0.1–100 mW/kg[1]. Hence, the SAR outside the head is substantially below 0.1 mW/kg. We had previously hypothesized that the mechanism of action of electromagnetic fields amplitude-modulated at insomnia-specific frequencies was due to modification in ions and neurotransmitters[6], as demonstrated in animal models[16], as such biological effects had been reported at comparable SARs. However, this hypothesis does not provide a satisfactory explanation for the clinical results observed in patients with advanced cancer. First, the levels of electromagnetic fields delivered to organs such as the liver, adrenal gland, prostate and hip bones, are substantially lower than the levels delivered to the head. Second, there is currently no acceptable rationale for a systemic anti-tumor effect that would involve subtle changes in neurotransmitters and ions within the central nervous system. Consequently, we hypothesize that the systemic changes (pulse amplitude, blood pressure, skin resistivity) observed while patients are exposed to tumor-specific frequencies are the reflection of a systemic effect generated by these frequencies. These observations suggest that electromagnetic fields, which are amplitude-modulated at tumor-specific frequencies, do not act solely on tumors but may have wide-ranging effects on tumor host interactions, e.g. immune modulation. The exciting results from this study provide a strong rationale to study the mechanism of action of tumor-specific frequencies *in vitro *and in animal models, which may lead to the discovery of novel pathways controlling cancer growth.

## Competing interests

AB and BP have filed a patent related to the use of electromagnetic fields for the diagnosis and treatment of cancer. AB and BP are founding members of TheraBionic LLC.

## Authors' contributions

BP and AB conceived and designed the study. FB and NK designed the device and performed the EM dosimetry. AB, BP and FC collected and assembled the data. BB and RF independently reviewed the imaging studies. AB, BP and FC analyzed and interpreted the data. BP wrote the manuscript. All co-authors read and approved the final manuscript.
